# Effects of Irradiation Combined with Partial Freezing Treatment on Texture, Flavor Compounds, and Storage Quality of Fermented Stinky Sea Bass

**DOI:** 10.3390/foods14061035

**Published:** 2025-03-18

**Authors:** Hong Xiao, Tingyu Feng, Hongying Liu, Yong Xue, Changhu Xue

**Affiliations:** 1 College of Biological and Food Engineering, Hubei Minzu University, Enshi 445000, China; 2024021@hbmzu.edu.cn; 2 Department of Food Science and Engineering, Ocean University of China, Qingdao 266003, China; xuech@ouc.edu.cn; 3 Qingdao Institute of Marine Bioresources for Nutrition and Health Innovation, Qingdao 266100, China; fengty999@sina.com; 4 College of Food Science and Technology, Hebei Agricultural University, Baoding 071000, China; liuhy@hebau.edu.cn; 5 Pilot National Laboratory for Marine Science and Technology (Qingdao), Qingdao 266003, China

**Keywords:** stinky sea bass, irradiation, partial freezing, flavor compounds, free amino acids

## Abstract

The preservation of fermented stinky sea bass (FSSB) has always been a major challenge. In this study, four preservation methods, including partial freezing (PF), freezing (F), irradiation + partial freezing (IPF), and irradiation + freezing (IF), were employed, and their effects on the storage quality of vacuum-packed fermented stinky sea bass were investigated. The results revealed that histamines and coliforms were not detected in any of the four preservation methods. Meanwhile, the TVB-N, peroxide value, pH, and cooking loss showed an increasing trend, while the hardness, springiness, and chewiness showed a decreasing trend, following FSSB vacuum packaging during the 30-day preservation period. Compared with PF or F only, irradiation significantly reduced the total plate number, TVB-N, pH, L*, and whiteness of the FSSB samples but increased the peroxide value content while maintaining the cohesiveness, gumminess, and chewiness. Furthermore, the of 1-butanol, propanal, and (E)-2-pentenal contents increased. In contrast, IPF preservation showed a better ability to maintain the texture quality of the FSSB. The safety index of the FSSB samples complied with the Chinese Standard GB10136-2015 National Food Safety Standards Animal Aquatic Products, following the 30-day preservation period under the four preservation methods. Overall, the experimental results suggest that the IPF preservation method is the most preferable approach to preserving FSSB, which can significantly maintain the product quality and save energy.

## 1. Introduction

Sea bass (*Lateolabrax japonicus*) stands as a highly profitable commercial fish widely cultivated in China, Thailand, the Pacific and Indian Oceans, and the Middle East. Its delicate white flesh and little bone spurs, as well as high protein and unsaturated fatty acid contents, align perfectly with contemporary dietary needs [1]. According to the “China Fishery Statistical Yearbook”, sea bass aquaculture production in China surpassed 180,000 tons in 2019 [2]. The current industrial sea bass processing mostly focuses on primary processing, such as salting, drying, and smoking [3,4]. The most common processed forms are salt-dried sea bass [5] and dry-cured sea bass [6]. However, the industrial processing of sea bass is significantly limited, posing a challenge that warrants immediate attention. To address this concern, fermented stinky sea bass was developed in our previous study [7,8]. However, the pre-experimental results showed that fermented stinky sea bass could only be preserved at room temperature for about 8 days after vacuum packing (TVB-N ≥ 30 mg/100 g), highlighting the significance of finding effective solutions to resolve its preservation problem.

Partial freezing refers to controlling the storage temperature of the product within a temperature range of 1–2 °C below its freezing temperature, typically ranging from −1 to −4 °C depending on the specific product [9]. It produces only a minimal number of ice crystals, resulting in minimal cell damage, reduced juice loss, and minimal changes in fish texture, which helps to preserve the unique flavor of the fish. Additionally, partial freezing consumes less energy and is more cost-effective, potentially extending the shelf life of the product by 1.5–4 times that of frozen products [10]. The primary advantage of using partial freezing technology is its ability to combine the beneficial effect of low temperature while converting some free water into ice, thereby reducing water spoilage [11]. Moreover, microbial activity is inhibited, and most bacteria are unable to grow at partial freezing temperatures [12]. However, it is worth noting that while microbial growth is inhibited, autolytic, enzymatic, or other chemical reactions may occur at a faster rate [13] or even accelerate, as observed by Magnussen et al. (2008) [14]. At present, partial freezing technology has been widely used for preserving muscle-building foods, including Atlantic cod (*G. morhua*), Atlantic salmon (*Salmo salar*), common carp (*Cyprinus carpio*) surimi, rabbit meat, buffalo meat steak, and boneless pork roasts [10].

Irradiation preservation involves the use of ionizing radiation for the ray treatment of food to achieve corrosion and sterilization, which extends the shelf life of the product. Irradiation has strong ray penetration, allowing for the sterilization of packaged food, which helps to prevent the secondary contamination of food packaging. Additionally, it results in minimal temperature changes during the sterilization process, minimal loss of nutritional value, and significant energy savings [15,16]. As such, irradiation has emerged as a viable method for the sterilization and preservation of aquatic products [17,18,19], as clearly defined by the Chinese Standard (NY/T 1256-2006) (2006) frozen aquatic product irradiation sterilization process standards [20].

Recently, there has been growing consumer interest in fermented stinky fish as a specialty, such as canned herring, stinky mandarin fish, and stinky sea bass. The prevailing preservation methods for fermented stinky fish mainly include freezing and ultra-high pressure [21]. However, the combination of irradiation with partial freezing has received less attention, and there is a lack of research on the preservation quality of fermented stinky fish by irradiation combined with partial freezing. Therefore, in this study, the stinky sea bass was taken as the research object, and the preservation effect of irradiation treatment, followed by partial freezing and freezing, on fermented stinky fish was investigated. Overall, the findings of this study could serve as a valuable resource for the preservation of fermented stinky fish.

## 2. Materials and Methods

### 2.1. Materials

Fresh sea bass (*Lateolabrax japonicus*) was purchased from the Chengyang Live Fresh Aquatic Products Wholesale Market (Qingdao, China). The sea bass were dead at the time of purchase and the experiments did not involve a slaughter process. Salt, Chinese prickly ash, and paprika were obtained from the Liqun Mart, Shaoxing, Zhejiang Province. Xian Hen stinky tofu (XH) was purchased online. The histamine standards were provided by Shanghai Maclean Biochemical Technology Co. (Shanghai, China). The 0.1001 mol/L sodium thiosulfate standard solution was provided by the Beijing North Weiye Measurement Technology Research Institute. The remaining chemicals and solvents were of analytical grade.

### 2.2. FSSB Sample Preparation

Fifteen fresh sea bass samples, each with an average weight of 950 ± 50 g, were gutted, had their gills removed, and were cleaned thoroughly. Based on the total mass of the sea bass, a combination of curing ingredients for dry curing was prepared with 4% (*w*/*w*) salt, 0.25% (*w*/*w*) Chinese prickly ash, and 0.25% (*w*/*w*) paprika. The fermented stinky sea bass (FSSB) samples were processed according to the method reported by Xiao et al. (2023) [7]. Finally, the FSSB were obtained and vacuum-packed for the subsequent irradiation and storage studies.

### 2.3. Irradiation Dose Selection Criteria and Experimental Design

According to the NY/T 1256-2006 frozen aquatic product irradiation sterilization process standards [20], the minimum effective dose should be 4 kGy, the highest tolerated dose should be 7 kGy, and the irradiation process dose should be 4–7 kGy, which would then combined with the actual experience of aquatic product irradiation production and processing. In this study, the commonly used irradiation dose of 6 kGy was selected. According to the actual sales needs, FSSB should be able to meet the shelf-life requirement of at least 30 days. Therefore, the shelf life was set to 30 days. The FSSB samples were irradiated with 6 kGy at the Huangdao Branch of Qingdao Blue Float Electron Irradiation Technology Co., Ltd. (Qingdao, China) and then preserved under partial freezing (−4 °C) and freezing (−18 °C). The non-irradiated group was set up for preservation under partial freezing (−4 °C) and freezing (−18 °C). The four experimental groups were preserved for 30 days, and the freshly produced FSSB samples were used as the control group for analysis. The samples preserved with the different preservation methods were labeled as follows: (1) partial freezing (−4 °C), labeled as PF; (2) freezing (−18 °C), labeled as F; (3) 6 kGy irradiated + partial freezing (−4 °C), labeled as IPF; (4) 6 kGy irradiated + freezing (−18 °C), labeled as IF; and (5) fresh fermented stinky sea bass, labeled as RW. The four groups of samples were removed from the 30-day storage and thawed at 4 °C for 12 h. Then, the surface of the vacuum bag was disinfected with 75% alcohol and the bag was immediately unwrapped with a sterilized scalpel on an ultra-clean bench. Later, the samples were subjected to microbiological index determination, and the stinky tofu was washed, cleaned, and dried using absorbent paper. Afterward, the samples from the same part were taken to determine the texture, and the remaining samples were removed from the head and tail, crushed with a grinder, and then stored at −80 °C for other index determination.

### 2.4. Total Volatile Basic Nitrogen (TVB-N) Analysis

The TVB-N was calculated using the Chinese Standard (GB 5009.228-2016) (2016) [22]. Briefly, 10 g of the crushed FSSB samples was precisely weighed. Then, 75 mL of distilled water was added to the sample, which was agitated to allow for liquid dispersion. After macerating for 30 min, 1 g of magnesium oxide was added, and the sample was immediately connected to a distiller. The determination was performed using an automatic Kjeldahl nitrogen analyzer.

### 2.5. Histamine Content Analysis

The histamine content was calculated using the Chinese Standard (GB 5009.208-2016) (2016) [23]. Water was used as a reference for measuring the absorbance at 480 nm. The standard curves of absorbance and histamine content (μg) obtained by the histamine standard were Y = 0.0048X + 0.003, r = 0.997.

### 2.6. Peroxide Value Content Analysis

The peroxide values of the FSSB were determined using the Chinese Standard (GB 5009.227-2016) (2016) [24].

### 2.7. Determination of Aerobic Plate Count

According to the national standard, GB 4789.2-2022 (Food Safety National Standard—Food Microbiological Test: Determination of Total Bacterial Colony) [25], the analysis was performed with slight modifications. Approximately 3 g of the sample was mixed with 27 mL sterile saline, followed by a 10-time dilution.

### 2.8. Coliform Determination

The coliforms in the various FSSB were determined according to the national standard, GB 4789.3-2016 (Food Safety National Standard—Food Microbiological Test: Coliform Count) [26], with minor modifications. Approximately 3 g of the sample was mixed with 27 mL sterile saline, followed by a 10-time dilution.

### 2.9. Texture Profile Analysis (TPA)

The TPA was performed using a texture analyzer (TMS-Pro, Food Technology, Inc., Sterling, VA, USA). The FSSB samples were taken from identical anatomical locations of the fishes and cut into 2 cm × 1.5 cm × 1.5 cm cubes. The texture profile analyses were carried out in accordance with the method described by Xiao et al. (2021) [27]. The trigger force was adjusted at 0.05 N, and the sea bass samples were pressed at a steady pace of 144 mm/min to 50% of their initial thickness. The distance between the texturometer’s probe and the sample base was adjusted to 40 mm.

### 2.10. pH Analysis

After homogenization in 27 mL of distilled water, the pH of each 3.0 g sample of FSSB meat was evaluated using a digital pH meter.

### 2.11. Color Analysis

The color of the sea bass samples was determined using a HunterLab ColorQuest XE, according to the method reported by Pathare et al. (2013) [28], and the results were represented as L* (brightness), a*, and b*. The whiteness index was calculated using the following equation:

(1) $$Whiteness=100-[{{\left(100-L*\right)}^{2}+{a*}^{2}+{b*}^{2}]}^{1/2}$$

### 2.12. Cooking Loss Analysis

The cooking loss analysis was performed according to the method reported by Xiao et al. (2021) [27]. Approximately 5 g of the sample was taken and steamed in boiling water for 10 min. The water on the surface of the fish was absorbed with paper, and the weight after steaming was measured; the cooking loss was calculated as follows:

(2) Cooking loss=(Weight initial−Weight final)/Weight initial×100%

### 2.13. Free Amino Acid (FAA) Analysis

Samples of precisely 8 g of crushed, skinless sea bass meat were weighed. Then, 15 mL of diluted hydrochloric acid at a concentration of 0.02 M was added to each sample, which was then vigorously homogenized. The sample was ultrasonically sonicated for 5 min (40–100 kHz, 20 °C), followed by a 20 min centrifugation at 4 °C and 8520× *g*. The supernatant was then transferred to a 50 mL volumetric flask, and then 10 mL of a 0.02 M hydrochloric acid solution was added to it. Later, the mixture was completely homogenized before being subjected to ultrasonic sonication for 5 min and centrifugation at 4 °C and 8520× *g* for 20 min. Subsequent experimental analysis was performed as described by Bi et al. (2022) [29].

### 2.14. GC-IMS Analysis of FSSB

Briefly, 2 g of crushed, skinless sea bass meat were placed in a 20 mL headspace container and sealed. According to the approach of Xiao et al. (2023) [7], the volatile organic components (VOCs) were examined using a gas chromatography–ion migration spectroscopy (GC-IMS) flavor analyzer (FlavourSpec^®^, Dortmund, Germany). The n-ketones C4–C9 (Sinopharm Chemical Reagent Beijing Co., Ltd., Beijing, China) were used as external references to compute the retention index (RI) of each volatile molecule. The VOCs were evaluated with a laboratory analytical viewer (LAV) and GC-IMS Library Search (FlavourSpec^®^).

### 2.15. Statistical Analysis

Each sample was tested in triplicate. The findings were examined using the SPSS statistical analysis software, version 16.0 (SPSS Inc., IBM, Armonk, NY, USA). A comparative analysis of the means was performed using one-way ANOVA combined with Tukey’s test. Wet weight was used as the unit of measurement for all data.

## 3. Results and Discussion

### 3.1. TVB-N Analysis of FSSB

**Figure 1:**
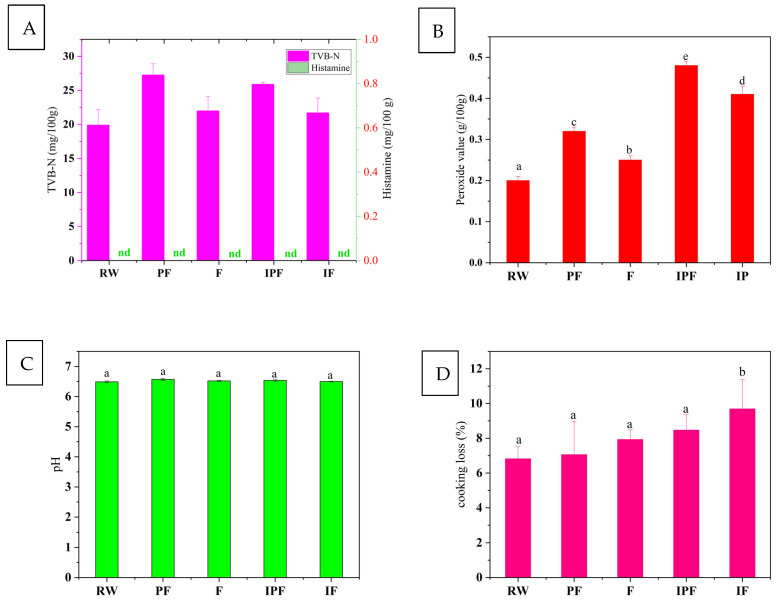
TVB-N, histamine, peroxide value content, pH, and cooking loss of FSSB under different preservation methods. (**A**) TVB-N and histamine content of FSSB under different preservation methods. (**B**) Peroxide value content of FSSB under different preservation methods. (**C**) pH of FSSB under different preservation methods; (**D**) cooking loss of FSSB under different preservation methods. Note: nd stands for no detection of the substance. RW: fresh fermented stinky sea bass; PF: partial freezing (−4 °C) preserved; F: freezing (−18 °C) preserved; IPF: 6 kGy irradiated + partial freezing (−4 °C) preserved; IF: 6 kGy irradiated + freezing (−18 °C) preserved.

According to the pre-experiment, after the FSSB were vacuum-packed and stored at room temperature, their TVB-N reached 30.6 mg/100 g on the 8th day, which exceeded the limit of 30 mg/100 g for the national standard, GB 10136 (Animal Aquatic Products) [30]. The TVB-N is an important indicator for determining the freshness of fish [31]. The formation of alkaline and nitrogen-containing compounds during the degradation of fish through enzyme activity and microbes causes a steady increase in TVB-N during fermentation [32]. As shown in Figure 1A, the lowest TVB-N content in RW was 19.9 ± 2.30 mg/100 g, while the freezing and irradiation + freezing after vacuum packaging were favorable to the suppression of TVB-N production in the FSSB samples. The TVB-N content of the samples preserved with irradiation + freezing was 21.70 ± 2.17 mg/100 g, which was better than freezing. Among the four preservation methods, the highest TVB-N content of 27.27 ± 1.65 mg/100 g was observed in the samples preserved with partial freezing, but it did not exceed the limit of 30 mg/100 g of the national food safety standard, GB10136-2015 (Animal Aquatic Products) [30].

In a study by Feng (2016) [33], tilapia fillets preserved with air conditioning combined with partial freezing showed that the TVB-N content increased during storage. On day 32, the TVB-N content of the tilapia fillets exceeded 20 mg/100 g, and the fish became inedible. The results of the partial freezing preservation of grass carp showed that the TVB-N content of the grass carp stored under microfrozen conditions for 21 days was 15.52 mg/100 g. The results of the preservation effect of Thunnini under different refrigeration temperatures showed that the TVB-N content of tuna stored under microfrozen conditions for 5 days was 10.88 mg/100 g. The above results indicate that partial freezing is an effective approach to preserving fish, and the changes in the TVB-N content of fish under partial freezing preservation conditions might be related to the fish species. In this study, the TVB-N content of RW reached 19.9 mg/100 g, probably because, after the pre-fermentation process, the endogenous protease of the fish meat decomposed the fish proteins and deaminated them to produce alkaline nitrogenous substances such as nitrogen, ammonia, and amines by the action of enzymes and microorganisms, causing the TVB-N content to increase. The TVB-N content increased relatively slowly during the preservation period, likely due to the addition of salt in the curing and fermentation processes during the pre-processing, inhibiting the growth of some microorganisms during preservation. Therefore, the increase in TVB-N was relatively slower than that of fresh fish, and the low temperature and irradiation inhibited the growth of microorganisms. Studies have shown that the effect of irradiation on TVB-N might be two-sided. For instance, during irradiation, the high-energy electron beam generated by the gas pedal can directly ionize or excite the molecules and atoms of the substance thus causing a cross-linking or polymerization of the macromolecules, resulting in protein damage, or indirectly ionizing the water molecules, resulting in free radicals and active factors that accelerate protein damage. Therefore, it can be inferred that although irradiation may lead to an increase in the TVB-N content, it can inhibit the growth and reproduction of microorganisms and cause the passivation of enzymes [17,34].

### 3.2. Histamine Content Analysis

According to the Chinese Standard (GB10136-2015) for animal aquatic products [30], the histamine levels in salted fish must not exceed 20 mg/100 g. In the presence of microorganisms, the amino acids in fish tissues are catalyzed by decarboxylase enzymes and undergo decarboxylation processes, resulting in histamine synthesis. Histamines are the most poisonous biogenic amine in fermented foods, reducing the quality of products that may be hazardous to human health (Liu et al., 2021) [35]. In this study, the safety of FSSB under four different preservation methods was confirmed by determining the histamine content of the five samples, and the results are shown in Figure 1A. The results showed that histamines were not detected in the FSSB meat of all five samples. The histamine content in all five samples did not exceed the national standard, GB 10136 (Animal Aquatic Products) [30]. Therefore, the histamine content of FSSB preserved for 30 days under the four preservation methods complied with the national standard for food safety.

### 3.3. Peroxide Value Content Analysis

During the curing and fermentation process, the body fat and other compounds in the FSSB undergo complicated chemical changes due to microbial actions or external circumstances such as temperature, humidity, and oxygen. Fat corruption occurs via two pathways, namely fat hydrolysis and fat oxidation. The peroxide value is a degree of measurement of fat oxidation. The peroxide value was measured to evaluate the preservation effect of the four preservation methods on the FSSB. As shown in Figure 1B, the peroxide value in the RW was 0.2 g/100 g, and the peroxide values in the PF, F, IPF, and IF groups were 0.32 g/100 g, 0.25 g/100 g, 0.48 g/100 g, and 0.41 g/100 g, respectively, which were consistent with the food safety national standard, GB10136-2015 (Animal Aquatic Products) [30], reporting that the peroxide content in salted fish must not exceed 2.5 g/100 g. The peroxide content of the F group was higher than that of the PF group, probably because the low temperature is not conducive to fat oxidation, and irradiation significantly increased the peroxide content in the preserved FSSB. Previous studies have shown that irradiation can induce accelerated lipid oxidation in sea bass meat, which is a typical reaction between free radicals and oxygen to produce lipid peroxide. Moreover, irradiation can catalyze the production of free radicals in fish meat thus accelerating lipid auto-oxidation to trigger free radical chain reactions [19]. Zhang et al. (2018) [36] studied the effect of electron beam irradiation on the sterilization and preservation quality of sea bass meat and found that electron beam irradiation could accelerate the lipid oxidation of sea bass meat. They also indicated that the higher the irradiation dose, the faster the oxidation rate, which is consistent with the present experimental results.

### 3.4. Determination of Total Bacterial Colony

**Figure 2:**
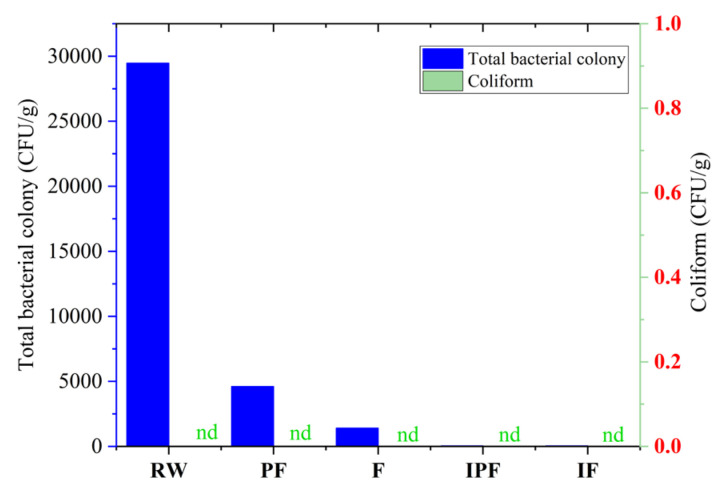
Total bacterial colony and coliform content of FSSB under different preservation methods. Note: nd stands for no detection of the substance. RW: fresh fermented stinky sea bass; PF: partial freezing (−4 °C) preserved; F: freezing (−18 °C) preserved; IPF: 6 kGy irradiated + partial freezing (−4 °C) preserved; IF: 6 kGy irradiated + freezing (−18 °C) preserved.

Microorganisms are important factors in fish processing and preservation, leading to changes in the quality of fish products. According to the NY/T 1256-2006 frozen aquatic products irradiation sterilization process standards [20], the total plate number of frozen aquatic products after irradiation must not exceed 5 × 10^4^ CFU/g. Similarly, the United States and European countries have specified that the total number of bacteria in aquatic products must not exceed 6log CFU/g. Therefore, it was necessary to determine the total plate number in the five samples. As shown in Figure 2, the total plate number in the five samples varied greatly, among which the total plate number in RW was 2.9 × 10^4^ CFU/g, and the total number of colonies in the PF group was 4.6 × 10^3^ CFU/g. Meanwhile, the total number of colonies in the F group was 1.4 × 10^3^ CFU/g and 33CFU/g in the IPF and IF groups, respectively. The total plate number in the samples of the irradiated group showed a significant decrease, indicating that 6 kGy irradiation could effectively kill the microorganisms in the FSSB. Compared with RW, both partial freezing preservation and freezing preservation reduced the total plate number in the samples, which might be due to the inhibition of microbial growth by vacuum packaging, followed by cryopreservation, which delayed the degradation of proteins by microorganisms and reduced the production of amines.

Zu et al. (2018) [37] studied the sterilization effect of irradiation on sea bass semi-finished products and found that the total number of bacterial colonies in sea bass semi-finished products decreased from 2500 CFU/g to <10 CFU/g after irradiation, which is consistent with the present experimental results. Zhang et al. (2018) [36] preserved sea bass through irradiation and found that the total plate number of sea bass meat on the first day of refrigeration was 3.48 log CFU/g, which was significantly higher than each irradiation group, and the total number of colonies decreased with the increasing irradiation doses. In this study, the total plate number in the samples decreased to 33 CFU/g after irradiation, followed by partial freezing preservation and freezing preservation, which might be due to the ionization and excitation of the bacterial cells under irradiation conditions, causing irreparable changes in cell function and reproduction and leading to microbial cell death or loss of reproductive capacity.

### 3.5. Coliform Determination

Coliforms are bacteria commonly found in the intestines of humans and warm-blooded animals, including *Escherichia coli*, *Citrobacter* spp., and *Klebsiella aerogenes*. During the process of food production, processing, storage, and transportation, irregularities in health management can be observed due to contamination by coliform bacteria. If the number of coliforms in aquatic products seriously exceeds the standards, it can destroy the nutritional composition of aquatic products, resulting in the loss of edible value and the accelerated spoilage of aquatic products. Therefore, coliform is an important microbial indicator for measuring the sanitary status of aquatic products. Consumption of aquatic products with excessive coliform bacteria may lead to diarrhea and, in severe cases, it may cause vomiting and other symptoms. According to the NY/T 1256-2006 standard for the irradiation sterilization process of frozen aquatic products [20], the coliform content of irradiated frozen aquatic products must not exceed 30 MPN/100 g. The coliform content of the five samples was determined, and the results are shown in Figure 2. As shown in Figure 2, coliforms were not detected in any of the five samples.

### 3.6. Texture Profile Analysis (TPA)

Hardness and chewiness determine the taste of fish. The effect of the four preservation methods on the texture of FSSB was investigated through TPA analysis, and the results are shown in Table 1. The hardness, springiness, and chewiness of the FSSB decreased under all four preservation conditions. Compared with RW, the hardness of the PF, F, IPF, and IF groups decreased by 11.20%, 8.72%, 20.37%, and 33.02%, respectively. Compared with partial freezing, freezing could better maintain the hardness, springiness, gumminess, and chewiness of FSSB. Compared with RW, the cohesiveness and gumminess of the FSSB in the IPF group increased by 25.00% and 1.27%, while the hardness, springiness, and chewiness decreased by 20.37%, 5.09%, and 3.82%, respectively. These results indicate that IPF preservation could better maintain the cohesiveness, springiness, gumminess, and chewiness of the FSSB. Compared with unirradiated processing, irradiated processing better maintained the cohesiveness, gumminess, and chewiness of the FSSB; however, IPF better maintained the textural quality of FSSB.

The texture of fish is significantly correlated with the fat and collagen contents of fish tissue, lethal method, storage time, and storage temperature. For instance, Taylor et al. (2002) [38] found that, in the early stage of ice storage, the textural changes were related to the disintegration of the cytoskeleton and the separation between muscle fibers; in the late stage of ice storage, the textural changes were related to the disintegration of connective tissues and the separation between muscle fibers and muscle septum. Que et al. (2015) [39] studied the effects of different preservation methods on the texture of *Channa argus* and found that the hardness decreased after 30 days of low-temperature storage (under −80 °C and −20 °C) and freezing and partial freezing conditions. The decrease in myogenic fiber salt-soluble protein content and protein denaturation within the muscle led to a decrease in muscle hardness, and the decrease in fish hardness might have been attributed to the decreased APT enzyme activity in fish, inducing actin denaturation. During the freezing process, the free water in the fish was frozen into ice crystals, which damaged the myocytes and induced changes in the stereological structure of the proteins, leading to decreased fish hardness [40].

### 3.7. pH Analysis

The pH changes in the FSSB under the four preservation methods were observed by measuring the pH. As shown in Figure 1C, the pH of the FSSB increased slightly under all four preservation conditions, with the highest pH increase being observed in the PF group. This result indicates that irradiation could inhibit the pH of FSSB during the preservation period. Li et al. (2023) [18] studied the storage quality of cooked silver carp cubes by electron beam irradiation and found that the pH of silver carp meat did not change significantly, between 6.2 and 6.9, at different storage temperatures, indicating little effect of storage temperature on pH. When the irradiation dose was 8 kGy, it effectively reduced the pH of the silver carp cubes during storage. This finding is consistent with the present study results.

### 3.8. Color Analysis

The color change in the FSSB under the four preservation conditions was observed by color measurement, and the results are shown in Table 2. As shown in Table 2, the L* value of RW was 65.90 and, at the end of preservation, the L* value decreased to 65.86 in the PF group and 65.51 in the F group. In contrast, irradiation, followed by microfreezing and freezing, further decreased the L* value of fish samples, but there was no significant difference in L* among the five samples. The whiteness of RW was 64.02, which decreased to 63.87 in the PF group and 63.57 in the F group as conservation proceeded, and irradiation exacerbated the decrease in whiteness of the samples. Zhang et al. (2018) [36] studied the change in color of the irradiated sea bass meat during refrigeration and found that the L* value of sea bass meat decreased from 45.94 to 43.87 as the preservation proceeded. Similarly, Zu et al. (2018) [37] studied the sterilization effect of irradiation on sea bass semi-finished products and found that the L* value of the sea bass semi-finished products decreased from 74.8 to 69.51 and the whiteness value decreased from 74.33 to 69.47 with 5.27–8.56 kGy irradiation, which is consistent with the present study results.

### 3.9. Cooking Loss Analysis

Cooking losses generally include the loss of water and soluble substances during the cooking process of the samples, and such cooking losses are mainly attributed to the structural damage of the muscle caused by the thermal denaturation of myogenic fibrous proteins. As shown in Figure 1D, the cooking loss of RW was only 6.82% and, after 30 days of storage, the cooking loss of the other four groups of samples increased from 7.06% in the PF group to 9.69% in the IF group. The cooking loss in the PF group was lower than in the F group, probably because at the partial freezing (−4 °C) temperature, the fish was in a partially frozen state, where some of the water could not be frozen, and thus the freezing damage was not as remarkable as freezing, and the fish protein was not easily denatured or only slightly denatured during microfreezing. On the contrary, during freezing, most of the water was in a frozen state, the protein was denatured, and the ice crystals gradually led to structural damage to the muscle tissue, making the juice loss of the fish samples more serious during steaming. After irradiation, the fish samples showed a trend of increasing cooking loss, but the difference was not significant (*p* > 0.05). The increased intensity of protein degradation might have hindered the contraction of myogenic fibers thus reducing juice loss and favoring the water-holding capacity of the meat. Meanwhile, irradiation was detrimental to protein hydrolysis and the water-holding capacity of fish, which in turn reduced the degradation of cytoskeletal proteins and increased steaming losses, respectively [41].

### 3.10. Free Amino Acid (FAA) Analysis

The production of FAAs during fermentation can increase the flavor and nutritional value of fermented fish [42]. FAAs generated from proteolysis contribute to the flavor and odor of fermented fish because they can be converted into volatile compounds using microbial enzymes via transamination and Hofmann elimination pathways [43]. The changes in free amino acid content in FSSB under the four preservation conditions were observed. Table 3 lists the 17 FAAs identified in the FSSB samples. Asp and Glu are MSG-like chemicals, which provide FSSB with an umami flavor, while Gly, Ser, Ala, Thr, Pro, and Arg provide a pleasant, sweet taste. Tyr, Lys, Met, Phe, Val, Ile, Leu, and His provide a bitter flavor [44]. Glu is a key umami-flavoring molecule in fish. As shown in Table 3, the contents of Glu acid and Asp decreased after 30 days of partial freezing or freezing storage, while the contents of Glu and Asp further decreased after irradiation, followed by partial freezing or freezing storage. The total umami amino acid decreased from 27.95 mg/100 g in the RW group to 16.15 mg/100 g in the IPF group; the total pleasant-tasting amino acid content decreased from 151.38 mg/100 g in the RW group to 142.56 mg/100 g in the IF group; the total free amino acid content increased to 242.47 mg/100 g in the IPF group.

Accumulating studies have reported that irradiation treatment could significantly increase the shelf life of meat products without causing any substantial changes in nutritional properties. For instance, the total amino acid content showed maintained and stable patterns, but post-irradiation storage increased or decreased the contents of essential and non-essential amino acids. Previous studies have shown a decrease in tyrosine, phenylalanine, and tryptophan in minced meat treated with doses ranging from 1 to 8 kGy e-beam [45]. Jo et al. (2018) [15] found a decreasing trend of Tyr content in smoked duck meat stored with increasing irradiation doses; however, compared with the control samples in the early stage of preservation, the contents of several amino acids such as Leu, Ile, His, and Ser were increased in the irradiated smoked duck meat. Lu et al. (2021) [17] studied the effect of different doses of electron beam irradiation on the flavor of sea bass and found that the total free amino acid content decreased with the increase in irradiation dose, while the total pleasant-tasting amino acid content decreased. In the present study, the contents of Gly, Ser, Thr, Lys, Met, His, and Cys were increased following irradiation. As for the physiological and functional activities of FAAs, the enzyme-triggered pathways are responsible for ionic equilibrium maintenance and protein solvation. According to Belitz et al. (2004) [46], glutamic and aspartic acids play important roles in these enzyme-triggered processes. The change in amino acid content might be related to the amino acid conversion by deamidation and transamidation caused by the free radicals generated by irradiation [47].

### 3.11. GC-IMS Analysis of FSSB

**Figure 3:**
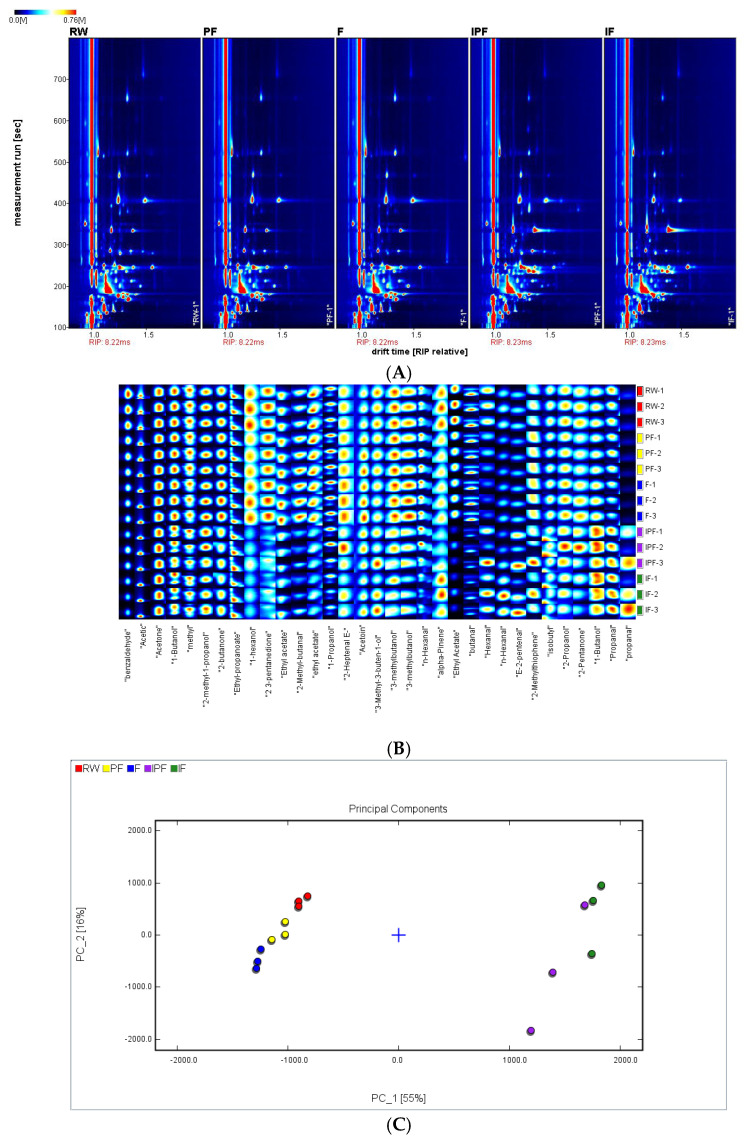
The GC-IMS analysis of FSSB under different preservation methods. (**A**) Two-dimensional GC-IMS spectra of characteristic flavor components of FSSB under different preservation methods. (**B**) The fingerprint of FSSB under different preservation methods. (**C**) Similarity analysis of FSSB under different preservation methods. RW: fresh fermented stinky sea bass; PF: partial freezing (−4 °C) preserved; F: freezing (−18 °C) preserved; IPF: 6 kGy irradiated + partial freezing (−4 °C) preserved; IF: 6 kGy irradiated + freezing (−18 °C) preserved.

The flavor substance changes in the FSSB under the four preservation conditions were explored, and the results are shown in Figure 3A. A total of 33 flavor substances were detected in the five fish samples, including 10 alcohols, 4 esters, 5 ketones, 10 aldehydes, and 4 other compounds (Figure 3B). Ethyl acetate, propan-2-ol, alpha-pinene, 2-methylbutanal, acetol, and 4-methylpentan-2-one have been reported as the primary characteristic flavor substances of FSSB [7]. The principal flavor ingredient in light-flavor Baijiu (GB/T 10781.2-2006) [48] is ethyl acetate. 2-propanol has an odor similar to a blend of ethanol and acetone. Since an FSSB lacks dimethyl disulfide, it has a distinct smelly flavor with a wine or ester flavor after cooking. In this study, ethyl acetate, 2-propanol, alpha-pinene, 2-methylbutanal, and other characteristic flavor substances of FSSB were detected in the five fish samples. The results of the PCA analysis revealed that RW was close to the PF and F groups and was located on the left side, indicating that the flavor substances of the FSSB samples did not change much in the later stages of partial freezing (PF) and freezing (F) preservation. However, the samples of the IPF, IF, RW, PF, and F groups were far away from each other. The irradiated samples are located on the right side in Figure 3C, all of which might have experienced a change in flavor substances after irradiation. Compared with the unirradiated samples, the contents of 1-butanol, propanal, and (E)-2-pentenal were increased in the irradiated samples (Figure 3B). Aldehydes have a low sensory threshold and, mostly, have aromatic characteristics such as clear, fruity, fatty, and nutty aromas. Ketones and alcohols are typically formed as a result of the oxidative breakdown of fatty acids. Their odor thresholds are often greater than aldehyde thresholds; however, their distinct floral, fruity, and other pleasant smells may contribute to distinct fish flavors [49]. Therefore, the increase in both alcohols and aldehydes after irradiation was beneficial to the flavor of the FSSB.

When the smoked duck meat was irradiated and then stored, the levels of acids and sulfur compounds in the flavor substances decreased with the increasing irradiation doses and storage periods, while the levels of alcohols, aldehydes, and phenols increased with increasing storage periods [15]. Zu et al. (2018) [37] studied the flavor effects of irradiation on semi-dried sea bass and found that benzene, alkanes, and alkenes were produced in the semi-dried sea bass after irradiation, and that 6.64 kGy irradiation increased the volatile components of alkenes such as tridecene and tetradecene in the semi-dried sea bass. It was found that the relative content of aldehydes and ketones in the sea bass increased after irradiation at different doses of electron beam irradiation. Compared with the control group, the content of aldehydes in the flesh samples of the sea bass increased from 21.97% to 23.38%, 26. 04%, 27.89%, and 29.81% after 1, 3, 5, and 7 kGy irradiations, respectively, which is consistent with the present study results [17]. The reason for the change in flavor of sea bass due to irradiation may be attributed to the direct action of an electron beam or the production of hydrogen by water irradiation and the indirect action of hydroxyl radicals on the fatty carbon chains of fish, which break the fatty carbon chains of fish, leading to the production of alkanes, olefins, and other volatile substances through lipid peroxidation and inducing the changes in fish flavors.

## 4. Conclusions

In this study, the effects of irradiation, freezing, and partial freezing on the preservation of FSSB were investigated to address the short shelf life of FSSB and contradict the fact that FSSB can only be preserved at room temperature for 8 days after vacuum packaging. The results showed that histamines and coliforms were not detected in all four preservation methods during the 30-day preservation period after vacuum packaging of FSSB but the TVB-N, peroxide value, pH, and cooking loss showed an increasing trend, while the hardness, springiness, and chewiness, as well as the total umami amino acids, of the FSSB showed a decreasing trend. Compared with partial freezing or freezing only, irradiation significantly reduced the total number of colonies, TVB-N, pH, L*, and whiteness of FSSB but increased the peroxide value and cooking loss while maintaining the cohesiveness, gumminess, and chewiness of FSSB. Meanwhile, the contents of flavor substances, including 1-butanol, propanal, and (E)-2-pentenal content, showed an increasing trend. Among the four preservation methods, irradiation + partial freezing preservation was the better method to maintain the textural quality. The safety indexes of FSSB after 30 days of preservation under the four preservation conditions conformed to the national food safety standard GB 10136 for animal aquatic products, which could extend the shelf life of FSSB from 8 days to 30 days. Overall, irradiation + partial freezing can be used as a potential approach for preserving FSSB under the premise of 30 days of preservation, which could maintain the product quality and save energy.

## Figures and Tables

**Table 1:** Texture profile analysis of FSSB under different preservation methods.

Samples	Hardness (N)	Cohesiveness	Springiness (mm)	Gumminess (N)	Chewiness (mJ)
RW	39.92 ± 3.32 ^b^	0.36 ± 0.01 ^a^	3.34 ± 0.85 ^b^	14.20 ± 1.58 ^b^	47.60 ± 14.03 ^b^
PF	35.45 ± 1.73 ^b^	0.38 ± 0.04 ^a^	2.35 ± 0.07 ^a^	13.59 ± 1.77 ^b^	31.99 ± 4.98 ^a^
F	36.44 ± 4.05 ^b^	0.38 ± 0.00 ^a^	2.40 ± 0.18 ^a^	13.90 ± 1.63 ^b^	33.60 ± 6.41 ^a^
IPF	31.79 ± 0.85 ^a^	0.45 ± 0.01 ^b^	3.17 ± 0.28 ^b^	14.38 ± 0.66 ^b^	45.78 ± 5.93 ^b^
IF	26.74 ± 1.70 ^a^	0.43 ± 0.04 ^b^	3.22 ± 0.70 ^b^	11.39 ± 0.57 ^a^	37.03 ± 10.06 ^a^

Note: Letters a and b in the same line indicate significant differences (*p* < 0.05) among them. Data are presented as the mean ± standard deviation (n = 3). RW: fresh fermented stinky sea bass; PF: partial freezing (−4 °C) preserved; F: freezing (−18 °C) preserved; IPF: 6 kGy irradiated + partial freezing (−4 °C) preserved; IF: 6 kGy irradiated + freezing (−18 °C) preserved.

**Table 2:** L*, a*, b*, and whiteness values of FSSB under different preservation methods.

Samples	L*	a*	b*	Whiteness
RW	65.90 ± 2.13 ^b^	−1.53 ± 0.78 ^a^	11.28 ± 0.87 ^b^	64.02 ± 1.93 ^b^
PF	65.86 ± 1.94 ^b^	−1.30 ± 0.75 ^a^	11.68 ± 0.69 ^b^	63.87 ± 1.82 ^b^
F	65.51 ± 2.15 ^b^	−1.26 ± 0.65 ^b^	11.51 ± 1.46 ^b^	63.57 ± 1.73 ^b^
IPF	63.74 ± 2.84 ^ab^	−0.65 ± 1.26 ^a^	9.80 ± 1.40 ^a^	62.37 ± 2.64 ^b^
IF	63.47 ± 2.63 ^a^	−1.37 ± 0.81 ^a^	11.52 ± 1.16 ^b^	61.62 ± 2.27 ^a^

Note: Letters a and b in the same line indicate significant differences (*p* < 0.05) among them. Data are presented as the mean ± standard deviation (n = 3). RW: fresh fermented stinky sea bass; PF: partial freezing (−4 °C) preserved; F: freezing (−18 °C) preserved; IPF: 6 kGy irradiated + partial freezing (−4 °C) preserved; IF: 6 kGy irradiated + freezing (−18 °C) preserved.

**Table 3:** Free amino acid content in FSSB under different preservation methods.

Items	Mouthfeel	RW	PF	F	IPF	IF
Glu	umami	25.47 ± 1.10 ^b^	19.00 ± 1.43 ^a^	20.15 ± 0.62 ^a^	14.71 ± 3.31 ^a^	15.66 ± 1.73 ^a^
Asp	umami	2.47 ± 0.58 ^a^	2.36 ± 0.05 ^a^	2.26 ± 0.65 ^a^	1.44 ± 0.04 ^a^	1.73 ± 0.34 ^a^
Gly	sweet	71.21 ± 1.45 ^a^	77.18 ± 9.80 ^a^	87.46 ± 11.18 ^a^	93.65 ± 5.69 ^a^	73.33 ± 9.35 ^a^
Ser	sweet	9.89 ± 0.23 ^a^	12.77 ± 0.73 ^b^	11.62 ± 1.30 ^ab^	13.78 ± 1.21 ^b^	10.91 ± 0.16 ^ab^
Ala	sweet	17.72 ± 0.62 ^a^	19.32 ± 0.85 ^a^	18.75 ± 1.14 ^a^	21.60 ± 6.29 ^a^	15.53 ± 1.25 ^a^
Thr	sweet	5.15 ± 0.69 ^a^	7.50 ± 1.00 ^a^	6.37 ± 0.35 ^a^	6.09 ± 0.41 ^a^	7.01 ± 1.15 ^a^
Pro	sweet	15.01 ± 0.35 ^a^	15.43 ± 3.66 ^ab^	15.67 ± 1.69 ^ab^	9.20 ± 0.11 ^a^	14.05 ± 0.76 ^a^
Arg	sweet	4.45 ± 1.41 ^ab^	5.24 ± 0.02 ^ab^	6.11 ± 0.64 ^b^	2.79 ± 0.40 ^a^	4.33 ± 0.87 ^ab^
Tyr	bitter	3.94 ± 1.03 ^a^	3.25 ± 0.18 ^a^	3.90 ± 1.25 ^a^	3.11 ± 0.35 ^a^	3.30 ± 0.25 ^a^
Lys	bitter	10.00 ± 3.27 ^a^	9.47 ± 1.36 ^a^	10.86 ± 0.41 ^a^	9.11 ± 1.54 ^a^	14.39 ± 2.14 ^a^
Met	bitter	2.99 ± 0.78 ^a^	3.74 ± 0.10 ^a^	3.60 ± 0.82 ^a^	4.06 ± 0.53 ^a^	3.64 ± 0.02 ^a^
Phe	bitter	9.80 ± 0.51 ^a^	9.71 ± 0.17 ^a^	9.97 ± 0.93 ^a^	6.81 ± 3.30 ^a^	4.93 ± 1.55 ^a^
Val	bitter	13.69 ± 4.06 ^a^	11.75 ± 0.51 ^a^	11.79 ± 0.70 ^a^	14.13 ± 2.33 ^a^	7.15 ± 1.61 ^a^
His	bitter	17.08 ± 0.21 ^a^	17.50 ± 0.89 ^a^	17.10 ± 2.54 ^a^	21.34 ± 3.41 ^a^	19.11 ± 0.35 ^a^
Ile	bitter	9.62 ± 0.36 ^a^	11.28 ± 0.49 ^a^	11.27 ± 0.85 ^a^	8.08 ± 3.69 ^a^	6.17 ± 1.68 ^a^
Leu	bitter	14.09 ± 0.70 ^a^	16.68 ± 0.72 ^a^	16.71 ± 1.58 ^a^	11.68 ± 5.53 ^a^	9.04 ± 2.72 ^a^
Cys	sour	0.00 ± 0.00 ^a^	0.00 ± 0.00 ^a^	0.00 ± 0.00 ^a^	0.89 ± 0.05 ^b^	0.81 ± 0.03 ^b^
Total umami amino acid	/	27.95 ± 1.69 ^b^	21.35 ± 1.48 ^a^	22.41 ± 1.27 ^ab^	16.15 ± 3.35 ^a^	17.39 ± 2.07 ^a^
Total pleasant tasting amino acid	/	151.38 ± 6.43 ^a^	158.81 ± 17.53 ^a^	168.37 ± 17.58 ^a^	163.26 ± 17.46 ^a^	142.56 ± 15.61 ^a^
Total FAA	/	233.14 ± 3.06 ^a^	242.19 ± 21.96 ^a^	254.24 ± 27.43 ^a^	242.47 ± 38.21 ^a^	211.08 ± 25.94 ^a^

Note: Letters a and b in the same line indicate significant differences (*p* < 0.05) among them. Data are presented as the mean ± standard deviation (n = 3). RW: fresh fermented stinky sea bass; PF: partial freezing (−4 °C) preserved; F: freezing (−18 °C) preserved; IPF: 6 kGy irradiated + partial freezing (−4 °C) preserved; IF: 6 kGy irradiated + freezing (−18 °C) preserved.

## Data Availability

The original contributions presented in the study are included in the article. Further inquiries can be directed to the corresponding author.

## References

[1] Li Q.Y., Zhou W.X., Zhang J.Y., Zhu J.S., Sun T., Li J.R., Cheng L. (2022). Synergistic effects of ε-polylysine hydrochloride and gallic acid on Shewanella putrefaciens and quality of refrigerated sea bass fillets. Food Control.

[2] Ministry of Agriculture and Rural Affairs of the People’s Republic of China, National Fisheries Technology Extension Center, China Society of Fishery (2020). China Fishery Statistical Yearbook.

[3] Fuentes A., Barat J.M., Fernandez-Segovia I., Serra J.A. (2008). Study of sea bass (*Dicentrarchus labrax* L.) salting process: Kinetic and thermodynamic control. Food Control.

[4] Oz F., Kotan G. (2016). Effects of different cooking methods and fat levels on the formation of heterocyclic aromatic amines in various fishes. Food Control.

[5] Fuentes A., Fernandez-Segovia I., Serra J.A., Barat J.M. (2010). Development of a smoked sea bass product with partial sodium replacement. LWT.

[6] Liu C., Zhang J., Wang Y. (2012). Lipolysis and Lipid Oxidation in Perch during Curing and Air Drying Ripening. Food Sci..

[7] Xiao H., Yu J., Hu M.Y., Liu H.Y., Yuan Z.Z., Xue Y., Xue C.H. (2023). Development of novel fermented stinky sea bass and analysis of its taste active compounds, flavor compounds, and quality. Food Chem..

[8] Xiao H., Feng T.Y., Yu J., Hu M.Y., Liu H.Y., Jiang X.M., Zhang T., Xue Y., Xue C.H. (2023). Development of room-temperature fermented stinky sea bass and novel insights into its physicochemical and flavor formation and microbial diversity. Food Biosci..

[9] Qin L.R., Wu Y.X., Chen J.W., Xia W.S., Liao E., Wang H.B. (2022). Effects of superchilling on quality of crayfish (*Procambarus clarkii*): Water migration, biogenic amines accumulation, and nucleotides catabolism. Int. J. Food Sci. Technol..

[10] Banerjee R., Maheswarappa N.B. (2019). Superchilling of muscle foods: Potential alternative for chilling and freezing. Crit. Rev. Food Sci..

[11] Kaale L.D., Eikevik T.M. (2014). The development of ice crystals in food products during the superchilling process and following storage, a review. Trends Food Sci. Tech..

[12] Kaale L.D., Eikevik T.M., Rustad T., Kolsaker K. (2011). Superchilling of food, a review. J. Food Eng..

[13] Einarsson H. (1988). Deep Chilling (Superchilling, Partial Freezing)—A Literature Survey.

[14] Magnussen O.M., Haugland A., Torstveit Hemmingsen A.K., Johansen S., Nordtvedt T.S. (2008). Advances in superchilling of food-process characteristics and product quality. Trends Food Sci. Technol..

[15] Jo Y., An K.A., Arshad M.S., Kwon J.H. (2018). Effects of e-beam irradiation on amino acids, fatty acids, and volatiles of smoked duck meat during storage. Innov. Food Sci. Emerg..

[16] Brewer M.S. (2009). Irradiation effects on meat flavor: A review. Meat Sci..

[17] Lu J.F., Zhu Y.K., Xu D.L., Zhang J.J., Huang T., Li C., Zhang H., Yang W.G. (2021). Effect of Electron Beam Irradiation with Different Doses on Flavor of *Lateolabrax japonicus* Meat. Food Sci..

[18] Li M.J., Huang J.J., Chen Y.N., Cai J., Li H.L., Zu X.Y. (2023). Storage Quality of Electron Beam Irradiated Cooked Silver Carp. Mod. Food Sci. Technol..

[19] Javanmard M., Rokni N., Bokaie S., Shahhosseini G. (2006). Effects of gamma irradiation and frozen storage on microbial, chemical and sensory quality of chicken meat in Iran. Food Control.

[20] (2006). Frozen Aquatic Product Irradiation Sterilization Process Standards.

[21] Wu Y.X., Wang Y., Yuan D.X., Zhang J.X., Chu L.M., Wang T.T., Jiang W., Sun H.J., Jin S.L. (2022). Effect of Ultra High Pressure on the Bacterial Community Structure and Quality of Stinky Mandarin Fish. Food Sci..

[22] (2016). National Food Safety Standard Determination of Volatile Salt Nitrogen in Food.

[23] (2016). National Food Safety Standard Determination of Biogenic Amines in Food.

[24] (2016). National Food Safety Standard Determination of Peroxide Value in Food.

[25] (2022). Food Microbiology Test-Determination of Aerobic Plate Count.

[26] (2016). Food Microbiology Test-Determination of Coliforms.

[27] Xiao H., Li N.N., Yan L.T., Xue Y. (2021). The Hydration Characteristics, Structural Properties and Volatile Profile of Squid (*Symplectoteuthis oualaniensis*) Mantle Muscle: Impacts of Steaming, Boiling and Sous-vide Cooking. Foods.

[28] Pathare P.B., Opara U.L., Al-Said F.A. (2013). Colour measurement and analysis in fresh and processed foods: A review. Food Boiprocess Technol..

[29] Bi S.J., Xue C.H., Sun C., Chen L.P., Sun Z.K., Wen Y.Q., Li Z.J., Chen G.D., Wei Z.H., Liu H.Y. (2022). Impact of transportation and rehydration strategies on the physiological responses of clams (*Ruditapes philippinarum*). Aquac. Rep..

[30] (2015). National Food Safety Standards Animal Aquatic Products.

[31] Oyelese O.A., Sao O.M., Adeuya M.A. (2013). Acidity/rancidity levels, chemical studies, bacterial count/flora of fermented and unfermented silver catfish (*Chrysichthys nigrodigitatus*). Food Nutr. Sci..

[32] Weiner I.D., Mitch W.E., Sands J.M. (2015). Urea and ammonia metabolism and the control of renal nitrogen excretion. Clin. J. Am. Soc. Nephrol..

[33] Feng L.N. (2016). Research on Keep Fresh Tedmology of Tilapia Fillets by Partial Freezing Combined with Modified Atmosphere.

[34] Yang W.G., Xu D.L., Lou Q.M., Zhang J.J., Li C., Shi X.Y., Shi H.G. (2015). Effect of Electron Beam Irradiation on Sterilization and Preservation of Fresh *Ostrea Plicatula*. J. Chin. Inst. Food Sci. Technol..

[35] Liu J.G., Lin C.X., Zhang W., Yang Q., Meng J., He L.P., Deng L., Zeng X.F. (2021). Exploring the bacterial community for starters in traditional high-salt fermented Chinese fish (Suanyu). Food Chem..

[36] Zhang H., Lv M.C., Mei K.L., Lu J.F., Yang W.G. (2018). Effects of Electron Beam Irradiation on the Preservation and Quality of Sea Bass (*Lateolabrax japonicus*) Meat. Food Sci..

[37] Zu X.Y., Li H.L., Zhang J.M., Chen Y.X., Liao T., Xiong G.Q. (2018). Effects of Different Doses of Electron Beam Irradiation on Sterilization and Quality of Semi-finished Products of *Micropterus salmoides*. Storage Process.

[38] Taylor R.G., Fjaera S.O., Skjervold P.O. (2002). Salmon fillet texture is determined by myofiber-myofiber and myofiber-myocommata attachment. J. Food Sci..

[39] Que T.T., Zheng J.W., Chen S.G., Jiang Q.Q., Liu W.J., Ye X.Q., Hu Y.Q. (2015). Effect of Super-chilling and Frozen on the Meat Quality of Snakehead. J. Chin. Inst. Food Sci. Technol..

[40] Brady P.L., Hunecke M.E. (1985). Correlations of sensory and instrumental evalutions of rosat beeef texture. Food Sci..

[41] Kristensen L., Purslow P.P. (2001). The effect of ageing on the water-holding capacity of pork: Role of cytoskeletal proteins. Meat Sci..

[42] Zang J.H., Xu Y.S., Xia W.S., Regenstein J.M. (2020). Quality, functionality, and microbiology of fermented fish: A review. Crit. Rev. Food Sci..

[43] Ji C.F., Zhang J.B., Lin X.P., Han J., Dong X.P., Yang S., Yan X.M., Zhu B.W. (2017). Metaproteomic analysis of microbiota in the fermented fish, *Siniperca chuatsi*. LWT.

[44] Chen D.W., Zhang M. (2007). Non-volatile taste active compounds in the meat of Chinese mitten crab (*Eriocheir sinensis*). Food Chem..

[45] Guillén-Casla V., León-González M.E., Pérez-Arribas L.V., Polo-Díez L.M. (2010). Direct chiral determination of free amino acid enantiomers by two-dimensional liquid chromatography: Application to control transformations in E-beam irradiated foodstuffs. Anal. Bioanal. Chem..

[46] Belitz H.D., Grosch W., Schieberle P. (2004). Amino acids, peptides, proteins. Food Chemistry.

[47] Josephson D.B., Lindsay R.C., Stuiber D.A. (1987). Enzyrnichydroperoxide initiated effects in fresh fish. J. Food Sci..

[48] (2006). Mild Flavor Chinese Spirits.

[49] Xu Y.X., Bai X.T., Feng Y., Zhao H.L., Li X.P., Li J.R., Yi S.M., Xie J., Guo X.H. (2021). Changes of Flavor Compounds in Sea Bass during Steaming Process as Analyzed by Gas Chromatography-Ion Mobility Spectroscopy and Chemometrics. Food Sci..

